# Lost dwell time and cycler alarms in inpatient automated peritoneal dialysis at a tertiary care hospital

**DOI:** 10.1080/0886022X.2024.2408432

**Published:** 2024-10-01

**Authors:** Maria C. Browne, Nasha Elavia, Adrienne Flowers, Ákos Géza Pethő, Abutaleb A. Ejaz, Sarah Khan, Ami M. Patel

**Affiliations:** a Division of Nephrology, University of Maryland School of Medicine, Baltimore, MD, USA; b VA Maryland Health Care System, Baltimore, MD, USA; c Department of Internal Medicine and Oncology, Semmelweis University, Budapest, Hungary

**Keywords:** Automated peritoneal dialysis, cycler, slow drain, inadequate drain volume, APD

## Abstract

**Background and aims:**

Dwell time is a critical component of automated peritoneal dialysis (APD) prescription, the stage at which transmembrane mass and fluid transfer occur. Loss of prescribed dwell time (LDT) can negatively influence the efficiency of APD. We investigated the incidence of LDT and related causes using APD in the acute care setting at a tertiary care center.

**Methods:**

Retrospective analysis was conducted of all inpatients receiving APD treatments from 1 December 2021 to 1 June 2023. Patient demographics, comorbidities, laboratory, and treatment data were extracted from electronic medical records and a propriety database.

**Results:**

*N* = 235 cycler treatments completed by 32 patients were included for analysis. The total LDT per treatment exceeding 30 minutes and 60 minutes occurred in 27% and 20% of all treatments. LDT of more than 10 minutes per each cycle exchange occurred in 26%. Session disruptions were caused by slow out-flow (55%), inadequate drain volumes (32%), patient line occlusions (20%), and priming errors (23%). The slow flow alarm requiring user intervention was reported to occur in about one-third of all treatments (31%).

**Conclusion:**

There was significant LDT and inadequate drain volume seen in about one-quarter and one-third of all inpatient APD treatments respectively. This can impact solute clearance and ultrafiltration.  Slow flow alarms were the most prevalent and the leading cause of LDT followed by inadequate drain volume. Future studies are required to investigate measures to reduce slow drain and improve drain volume in the hospital setting.

## Introduction

The acceptance of peritoneal dialysis (PD) has increased due to the ease and flexibility of performing PD at home afforded with technological advancements in safety, customization, and portability of cyclers with automated peritoneal dialysis (APD). According to the US Renal Data System (USRDS), the percentage of PD patients choosing APD increased from 47% in 2000 to 80% in 2015 while the total PD proportion of all patients receiving dialysis merely increased from 8.9% to 10% during the same time period [1]. APD is considered superior to manual continuous ambulatory peritoneal dialysis (CAPD) in achieving ultrafiltration targets, particularly for patient with fast transporter membrane properties [2], and providing greater psychosocial well-being for patients and family members [3,4]. In the hospital environment, APD may have multiple advantages over CAPD including lower risk of peritonitis from fewer connections, increased treatment options, and potentially reduced nursing time in addition to patient’s preference and comfort [5,6].

Modern cyclers are equipped with multiple safety and comfort features which include intra-peritoneal pressure control, inflow/outflow rate sensor, and ability to vary volume and dwell duration [4]. Furthermore, newer innovations have made remote monitoring possible, which improves the real-time bidirectional communication to promote adherence, monitor treatment, and modify prescriptions timely, thereby reducing in-person visits [5]. Additional features such as touch-screen panel with animated graphics and voice guidance with automated instructions and troubleshooting has reduced training time [5]. A specialized 24/7 clinical helpline is available for additional support to the bedside nurses who may have limited exposure in operating the cycler [7,8].

Studies investigating the management and efficiency of PD in the acute care setting are lacking. Adequate toxin and fluid removal in PD are dependent on exposure (dwell) time, number of exchanges, dialysate fluid concentration, and peritoneal membrane kinetics. Dwell time is critical as the diffusion of toxins and fluids occur during the crucial dwell time phase and any problem with either inflow or outflow would lead to loss of dwell time from the prescribed total treatment time. The present study analyzes inpatient cycler treatments with the aim to report the frequency of lost dwell time (LDT) and various cycler alarms at a single tertiary care hospital using APD.

## Methods

This is a single-center retrospective study of end-stage-kidney disease (ESKD) patients, aged >18 years who were receiving APD during their hospitalization at the University of Maryland Medical Center from 1 December 2021 to 1 June 2023. APD treatments were provided using Baxter Amia^TM^ cycler machines. APD prescriptions were ordered by treating nephrologists *via* the dedicated, propriety software, Sharesource, and transmitted to the patient’s bedside cycler machine. All treatment data including alarms, session disruptions, ultrafiltration volumes, and adherence to prescription usually are uploaded back to the Sharesource allowing nephrologists to remotely review and adjust prescription. All treatments were time-based, and tidal PD was not performed. Session with incorrect APD orders and missing treatment flowsheets were excluded from the study. Demographic data, comorbidities, length of hospitalization, incidence of infectious complications (peritonitis, exit-site infection), catheter-related mechanical complications, use of intraperitoneal heparin and antibiotics, use of 4.25% Dianeal® solution, and incidence of transfer to hemodialysis were extracted from electronic medical records. Peritonitis was diagnosed with meeting at least two of the following criteria as defined by the International Society of Peritoneal Dialysis: (1) presence of abdominal pain and/or cloudy dialysis effluent, (2) dialysis effluent white cell count >100/µL with >50% polymorphonuclear leukocyte and (3) positive dialysis effluent culture [9].

Data on prescribed and actual completed number of cycles, prescribed and actual dwell times, frequency of cycler alarms, fill volumes, and ultrafiltration were extracted from Sharesource. Data are expressed as mean ± standard deviation unless otherwise stated. This study was approved by the University of Maryland, Baltimore Institutional Review Board (protocol: HP-00105990) and all methods were carried out in accordance with the guidelines and regulations.

### Definition of selected cycler alarms

There are various cycler alarms recognized by the Amia™ system. Recorded alarms selected for review in this study are outlined below:

- Slow flow alarms are referred to as Slow Flow Escalating Alert and Slow Flow User Alert within the Amia™ system. Slow Flow Escalating Alert is defined as persistent slow outflow drain below 50 ml/min for more than 2 minutes resulting in an audible alert beeping every 30 seconds. If the situation corrects itself, the escalating alert resolves. On the other hand, if the slow flow persists, the alarm intensifies to Slow Flow User Alert. The therapy is automatically paused and requires the user to intervene and respond to continue therapy. In this study, the Slow Flow User Alert is referred to as the s*low flow alarm requiring intervention*.
- *Inadequate drain volume alarm* occurs when the expected drain requirement is not met within 2 minutes of receiving the Patient Slow Flow Escalating Alert.
- *Patient line occlusion alarm* occurs when the patient line is occluded, which can happen if the transfer set is closed, patient line is kinked or twisted, patient line is occluded with fibrin plug, or cycler is more than 30 cm (12 in) above the catheter.
- *Priming error alarm* for this study consisted of alerts related to incomplete priming of either patient line or solution lines, which could be related to kinks, clamps, the frangible pin was not broken, or solution bag was not connected.
- *Unexpected volume alert* is defined as more than 150 ml during the initial drain when 50 ml or less was expected.
- *Drain line occlusion alert* occurs when the drain line is blocked, or cycler is more than 1 meter below the drain line outlet. Drain slow flow alert is related to slow flow in the drain line. Other causes related to drain line occlusion or slow flow are kinking of the lines or presence of fibrin [10].

## Results

### Demographics

Two hundred and forty-seven cycler treatments were reviewed, 12 flowsheets were excluded for missing treatment or incorrect orders, and the remaining 235 treatments were included for analysis. The 235 cycler treatments completed by 32 patients were included in the analysis. The mean age was 57.6 ± 15.2 years. Majority of the patients were Black (56.3%), White 25%, Asian 15.6% and 3.1% identified as other races. There were 20 males and 12 female patients. All but one patient had Baxter transfer set which connected to our hospital PD supplies. The one patient with Fresenius transfer set required adaptor and Baxter transfer set to connect to our PD supplies. More than half of the patients (62.5%) had residual renal function with documented daily urine output greater than 100 ml as per initial admission. The average length of hospitalization was 16.6 ± 19.1 days with hospitalization exceeding 10 days occurred in about one-third of the cases. The hospital diagnoses for patients exceeding 10-day hospital stay include stem-cell transplantation, cardiogenic shock, cardiac surgery, intracranial bleeding, and sepsis. Table 1 outlines the demographic details of the patients.

**Table 1.** Demographics of patients in the study population.

	Mean ± SD	*N* (%)
Age (years)	57.6 ± 15.16	
Race:		
Black		18 (56.3)
White		8 (25)
Asian		5 (15.6)
Other		1 (3.1)
Weight (kg)	84.28 ± 25.46	
Etiology of ESKD		
Hypertension		10 (31.2)
Diabetes		12 (37.5)
Both		2 (6.2)
Unknown		4 (12.5)
Patients with residual kidney function (urine output >100 ml/day)		20 (62.5)
Patients with HFrEF		12 (37.5)
Patients with HFpEF		2 (6.3)
Length of hospitalization (days)	16.6 ± 19.1	
Complications:		
Transfer to HD or CRRT		4 (12.5)
Peritonitis		3 (9.4)
Exit site infection		0
Surgical revision of PD catheter		1 (3.1)

HFrEF: heart failure with reduced ejection fraction; HFpEF: heart failure with preserved ejection fraction; HD: hemodialysis; CRRT: continuous renal replacement therapy; PD: peritoneal dialysis.

### Primary outcomes

**Figure 1.**
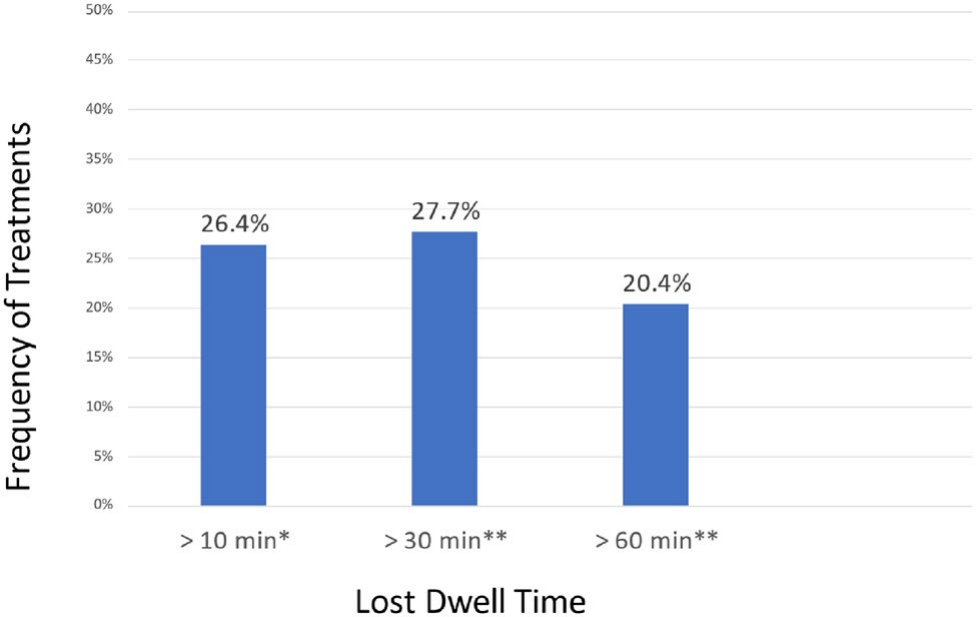
Frequency of lost dwell time. *Lost dwell time per cycle. **Lost dwell time per treatment

Table 2 summarizes the APD treatment data. The average number of cycles per treatment was 4.7, and the average fill volume per exchange delivered was 2270 ml. The overall usage of 4.25% Dianeal Low Calcium® solution was reported in only 4 treatments (1.7%). Most treatments utilized 2.5% and/or 1.5% Dianeal Low Calcium® solutions. The total LDT was calculated by the difference of the prescribed dwell time and delivered dwell time over the entire treatment. Approximately 27% and 20% of all treatments had total LDT exceeding 30 minutes and 60 minutes respectively (Figure 1). About 26% of treatments had more than 10 minutes LDT per each exchange cycle.

**Table 2.** APD prescription and treatment data.

Number of cycles	5 (3–8)
Fill Volume (ml)	2001 (1001–3001)
Night Ultrafiltration (ml)	592 (^−^301–2885)
Dwell Time Prescribed (min)	90 (47–170)
Dwell Time Performed (min)	88 (11–175)
Lost Dwell Time per Cycle (min)	0 (^−^9–132)
Total Lost Dwell Time* (min)	0 (^−^45–396)

Data displayed as median (minimum-maximum range).

*Denotes total dwell time lost during entire treatment.

**Figure 2.**
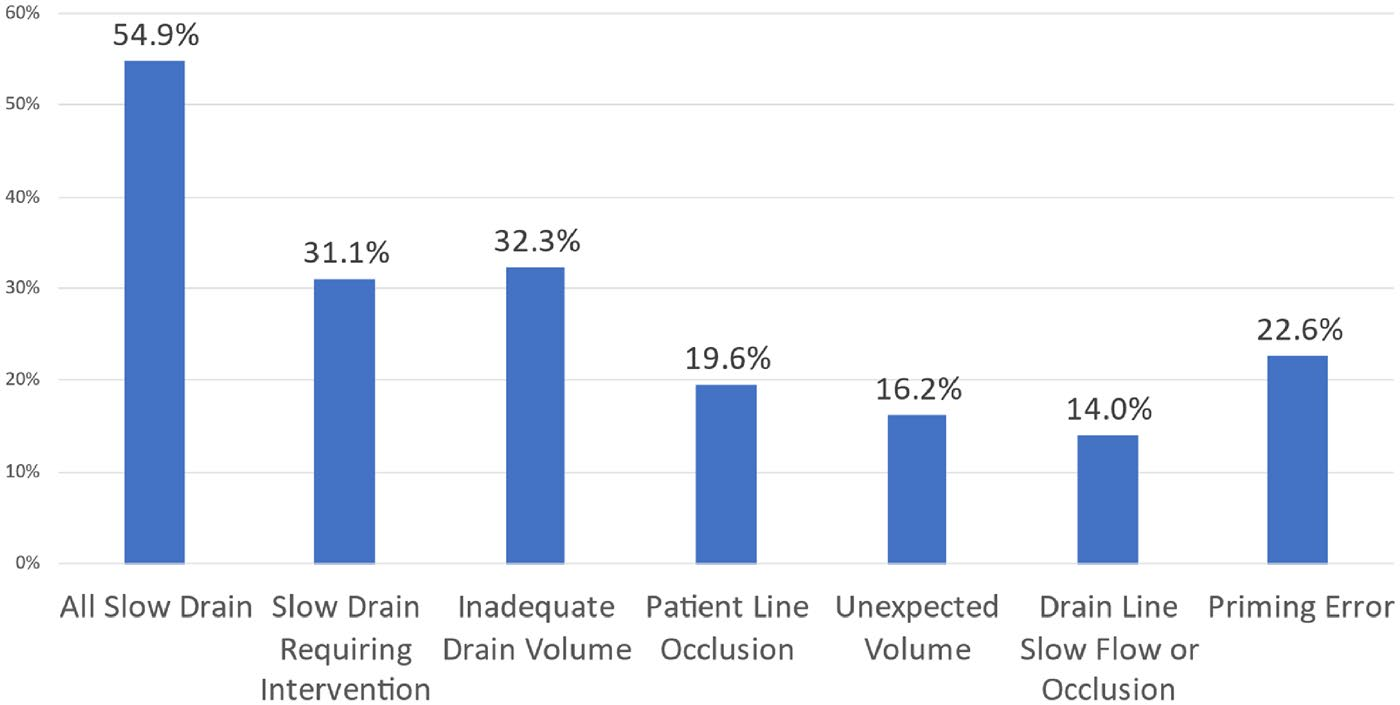
Prevalence of cycler treatments with selected cycler alarms.

**Figure 3.**
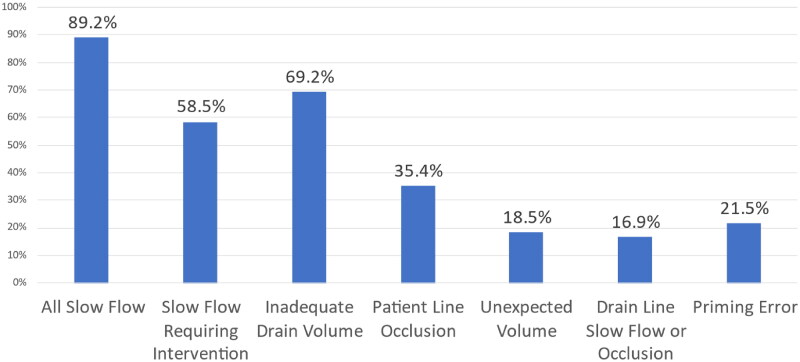
Frequency of selected alarms in cycler treatments exceeding 30 min lost dwell time.

Figure 2 depicts the proportion of treatments with various cycler alarms. The most common alarms were related to draining which consisted of slow flow, occurring in about half of all treatments (55%), followed by inadequate drain volume (32%), patient line occlusion (20%), and priming error (23%) alarms. The slow flow alarm requiring intervention was reported to occur in about one-third of all treatments (31%). In this cohort, the leading cause of total LDT exceeding 30 minutes was due to slow outflow drain (89%) followed by inadequate drain volume (69%) (Figure 3). If inadequate drain volume occurs, the Amia^TM^ system allows for an option of Smart Drain, by adding more rapid cycles using smaller volume to complete the treatment. A total of 9 treatments (3.6%) utilized the Smart Drain feature. Rare alarms include heater slow flow, solution slow flow, and AC power failure/restoration. Serious alarms occurred rarely. Maximum air alarm occurred in 1 treatment resulting in immediate termination.

### Secondary outcomes

Peritonitis occurred in 3 patients (9.4%) and 4 patients (12.5%) were switched to hemodialysis (HD) or continuous renal replacement therapy (CRRT). One patient was admitted with peritonitis with outpatient PD fluid culture growing *Bacteroides vulgaris*. The other two patients developed culture negative peritonitis with one arising from a perineal wound infection and the second from presumed bowel translocation from gastrointestinal bleed which later required emergent bowel resection and PD catheter removal. One patient had wet contamination and received 1 day of intravenous antibiotics with subsequent normal PD cell count and negative cultures. The 2 of the 3 patients who were transitioned to CRRT died in the hospital due to severe shock. One patient was converted to CRRT then HD due to inadequate PD treatments from delay draining after presenting with intracranial bleed, and PD catheter was removed. One patient (3.1%) required surgical revision of PD catheter due to dysfunctional catheter that occurred after catheter was not used for weeks following complicated cardiac surgery. Three patients (9.4%) received intraperitoneal heparin for prevention of fibrin clogging or presence of fibrin.

## Discussion

We report the incidence of lost dwell time and cycler alarms with APD in a tertiary academic medical center. The major finding was that almost a quarter of all treatment sessions were associated with LDT exceeding 30 minutes, and 20% of treatments had LDT exceeding 60 minutes. The leading cause of LDT was slow outflow drain, followed by inadequate drain volume and patient line occlusion. Slow drain alarm requiring nursing intervention occurred in about one-third of all treatments. An unexpected finding was the occurrence of priming error in 23% of the treatments, highlighting the need for increased training of the bedside nurses operating the cycler.

Cycler alarms are important inbuilt safety features that prevent serious patient adverse events such as overfilling, volume overload, pneumoperitoneum, in addition to identifying catheter malfunction [7]. Constipation is a frequent cause for catheter malfunction from mechanical extraluminal occlusion occurring from dilated bowel. In the hospital, the risk of constipation increases due to multitude of factors such as use of narcotics, lack of activity, decreased fluid intake, changes in diet and sleep quality, lack of privacy, and decreased access to toilet facilities [11]. Empiric use of laxatives upon hospitalization should be the first-line approach in preventing slow flow and inadequate drain alarms. Other causes of catheter malfunction include catheter migration, omental wrap, adhesions, fibrin plug, and kinking of the catheter [12]. In our study, most patients were prescribed laxatives, approximately 9% of patients received intraperitoneal heparin for the prevention or presence of fibrin, and one patient (3.1%) required surgical intervention of clogged PD catheter to restore patency after prolong PD rest exceeding 2 weeks.

The evidence of prophylactic flushing in the setting of PD rest to prevent PD catheter malfunction is lacking, and the intervals of flushing can vary from alternate daily to monthly [13]. We recommend once a week rapid exchange during PD rest to maintain catheter patency and to consider the use of heparin-containing PD solution for those patients at higher risk of fibrin clogging. Alteplase can also be used to clear fibrin-clogged PD catheter that is refractory and has been shown to restore catheter patency [14]. As last resort, surgical intervention or manipulation with fluoroscopic stiff wire for catheter reposition maybe required for selected refractory catheter dysfunction [14,15].

As APD has become the predominant mode for outpatient PD in the US, patients often prefer to continue APD in the hospital setting. There are multiple advantages of APD compared to CAPD in the hospital including lower risk of peritonitis from fewer connections, increased customization of treatment which can allow for urgent start PD, potentially reducing nursing time, and preferred modality for patients with rapid transport membrane characteristics. Furthermore, APD provides more details related to PD treatment such as precise ultrafiltration, dwell time, and presence of inflow and outflow issues. Although modern machines are equipped with animated graphics and voice guidance for cycler setup and troubleshooting alarms, unnecessary alarms can result in significant LDT causing inadequate clearance and fluid removal, as well as increased resource utilization, and contribute to poor quality of sleep and comfort.

The inherent limitations of this retrospective, single-center study include the small sample size and varied data collection with incomplete or inconsistently measured data. Correlation of LDT and ultrafiltration goals and adequacy of dialysis could not be analyzed due to unavailability of weekly Kt/V data. These measurements are not routinely performed in the hospital setting. In addition, there was no comparative cohort representing outpatient APD.

In conclusion, our study reported significant LDT and identified major cycler alarms of APD in the acute care setting. We identified areas of intervention to potentially reduce the incidence of cycler alarms, thereby improving the quality and efficiency of inpatient APD treatment. Some of these measures include preemptive use of laxatives, low threshold of utilizing intraperitoneal heparin, prophylactic catheter flushing in setting of PD rest, and improving PD training of hospital care team. Future well designed studies are required to investigate measures to reduce slow drain and improve drain volume in the hospital setting.  
